# T-cell immunoglobulin and mucin domain-containing protein 3–mediated immunomodulation in myeloid cells and keratinocytes in the development of severe acne

**DOI:** 10.1186/s43556-025-00367-3

**Published:** 2025-11-27

**Authors:** Siliang Chen, Xiaoyun Wang, Yidan Xu, Wanxin Zeng, Gu He, Xiang Wen

**Affiliations:** 1 Department of Dermatology and Venereology, West China Hospital, Sichuan University, Chengdu, 610041 China; 2 Laboratory of Dermatology, Clinical Institute of Inflammation and Immunology, Frontiers Science Center for Disease-Related Molecular Network, State Key Laboratory of Biotherapy, West China Hospital, Sichuan University, Chengdu, 610041 China

**Keywords:** Severe acne, TIM3, Single cell transcriptomics, Macrophages, Keratinocytes

## Abstract

Acne vulgaris is a common chronic inflammatory skin disorder with significant clinical and societal impacts. Severe acne (SA), in particular, causes scarring, disfigurement, and psychosocial distress. The underlying pathogenesis of SA remains poorly understood, hindering the development of effective treatments. In our study, single-cell RNA sequencing (scRNA-seq) of samples from SA, acne, and normal skin was performed, 12 clusters were identified from the total cell population, and macrophages were considered as important cell clusters since all gene modules showed high gene activity for macrophages in the scRNA weighted correlation network analysis (WGCNA). By integrating macrophage-specific differentially expressed genes (DEGs) with Mendelian randomization (MR) analysis, it was found that T-cell immunoglobulin and mucin domain-containing protein 3 (TIM3) was identified as a potential immune checkpoint involved in the development of SA. Multiplex immunohistochemistry (mIHC) revealed a decreased tendency for TIM3^+^ neutrophils, an increased presence of TIM3^+^ macrophages (especially TIM3^+^ M2 macrophages), and a reduced population of TIM3^+^ keratinocytes in SA tissues compared to controls. In human immortalized keratinocytes (HaCaT) cells, *TIM3* knockdown led to the upregulation of *Propionibacterium acnes (P. acnes)*-induced proinflammatory cytokine secretion, and the administration of an anti-Tim3 antibody in *P.acnes* induced mouse model exacerbated acne-associated inflammation. Collectively, these findings support a role for TIM3 in SA and suggest TIM3 as a potential therapeutic target, while underscoring the immunomodulatory function of keratinocytes and providing directions for future investigations.

**Supplementary Information:**

The online version contains supplementary material available at 10.1186/s43556-025-00367-3.

## Introduction

Acne vulgaris is among the most prevalent dermatoses globally, affecting approximately 9.8% of the population, with the highest incidence observed in adolescents and young adults [1], imposing a considerable global burden and contributing to substantial healthcare costs because of its chronic nature and the need for ongoing management. Severe acne (SA), in particular, is not merely a cosmetic concern but also a chronic inflammatory disorder with profound physical, psychological, and societal consequences [2–4]. SA frequently leads to scarring and disfigurement, which increases the risks of anxiety and depression and reduces quality of life [5]. In a population-based screening of 4,561 13-year-olds, moderate-to-severe acne affected 14% of girls and 9% of boys, values that translate into large absolute numbers when applied to entire cohorts [6], and a 2023 meta-analysis (24,649 patients) revealed a pooled acne-scarring prevalence of 47% [7].

Current research highlights the roles of hormonal imbalances, microbial dysbiosis, and immune responses, in the acne development [8–11]. However, the complex interactions between these factors at the cellular level remain inadequately understood. Existing treatments, including systemic isotretinoin, antibiotics and topical applications, are often combined but are limited by low patient adherence due to their complexity or side effects [12]. These limitations underscore the need for alternative targeted therapies, such as small molecular inhibitors and other innovative approaches, to address the intricate interplay of immunological, microbial, and inflammatory factors underlying acne pathogenesis. Although useful, traditional bulk sequencing methods fail to reveal the cellular heterogeneity and intricate molecular mechanisms underlying SA. Single-cell sequencing (scRNA-seq) enables unbiased profiling of thousands of individual cells, allowing the identification of distinct immune and non-immune cell populations within SA lesions that are obscured in bulk data [13]. Our study is to explore the SA development through scRNA analysis.

T-cell immunoglobulin and mucin domain-containing protein 3 (TIM3, whose official gene symbol is hepatitis A virus-cellular receptor 2, *HAVCR2*) is a member of the TIM family of immunoregulatory proteins [14], and was first discovered on interferon γ (IFNγ)-secreting T helper (Th) 1 and cytotoxic T (Tc) 1 cells, where it serves as a marker for T-cell exhaustion [15, 16]. Although TIM3 is well known for its role in regulating T-cell function, its involvement in myeloid cells has recently gained considerable attention [17, 18]. Similar to its function in T cells, TIM3 serves mainly as an inhibitory receptor in myeloid cells. Myeloid cells play important roles in acne development by responding to *Propionibacterium acnes (P. acnes)* and producing proinflammatory factors such as interleukin (IL)−1β, IL-6, IL-8, and tumor necrosis factor α (TNFα), which contribute significantly to acne inflammation [11, 19–21]. However, despite the established role of myeloid cells in acne development, whether TIM3 serves as an immune-checkpoint in myeloid cells and keratinocytes during SA development is not clear yet. The expression and function of TIM3 in SA are needed to be investigated.

In our study, *TIM3* has emerged as the key gene involved in SA, through an integrative approach combining scRNA-seq and Mendelian randomization (MR). The expression of TIM3 in macrophages, neutrophils, and keratinocytes was investigated by multiplex immunohistochemistry (mIHC) staining, after which, the functional role of TIM3 in SA pathogenesis was explored through in vivo and in vitro experiments. Inflammatory cytokines were upregulated after TIM3 was knocked down in human immortalized keratinocyte (HaCaT) cells. The interaction between TIM3 and Galectin 9 (GAL9) was also clarified in HaCaT cells through coimmunoprecipitation (Co-IP) experiments. In a mouse acne model, *P. acnes*–induced inflammation was amplified after intravenous injection of an anti-Tim3 antibody. Our results reveal that TIM3 is a putative immune checkpoint in keratinocytes and myeloid cells (macrophages and neutrophils) during SA, confirming a previously underappreciated role in SA pathogenesis. Prior work on TIM3 has focused largely on its checkpoint function in malignancies, with limited attention given to skin diseases such as SA. By extending this paradigm to nonmalignant cutaneous inflammation, our findings clarify a mechanistic axis relevant to SA and suggest that targeting TIM3 could inform the development of novel therapeutic strategies.

## Results

### Study design and sample characteristics

**Fig. 1:**
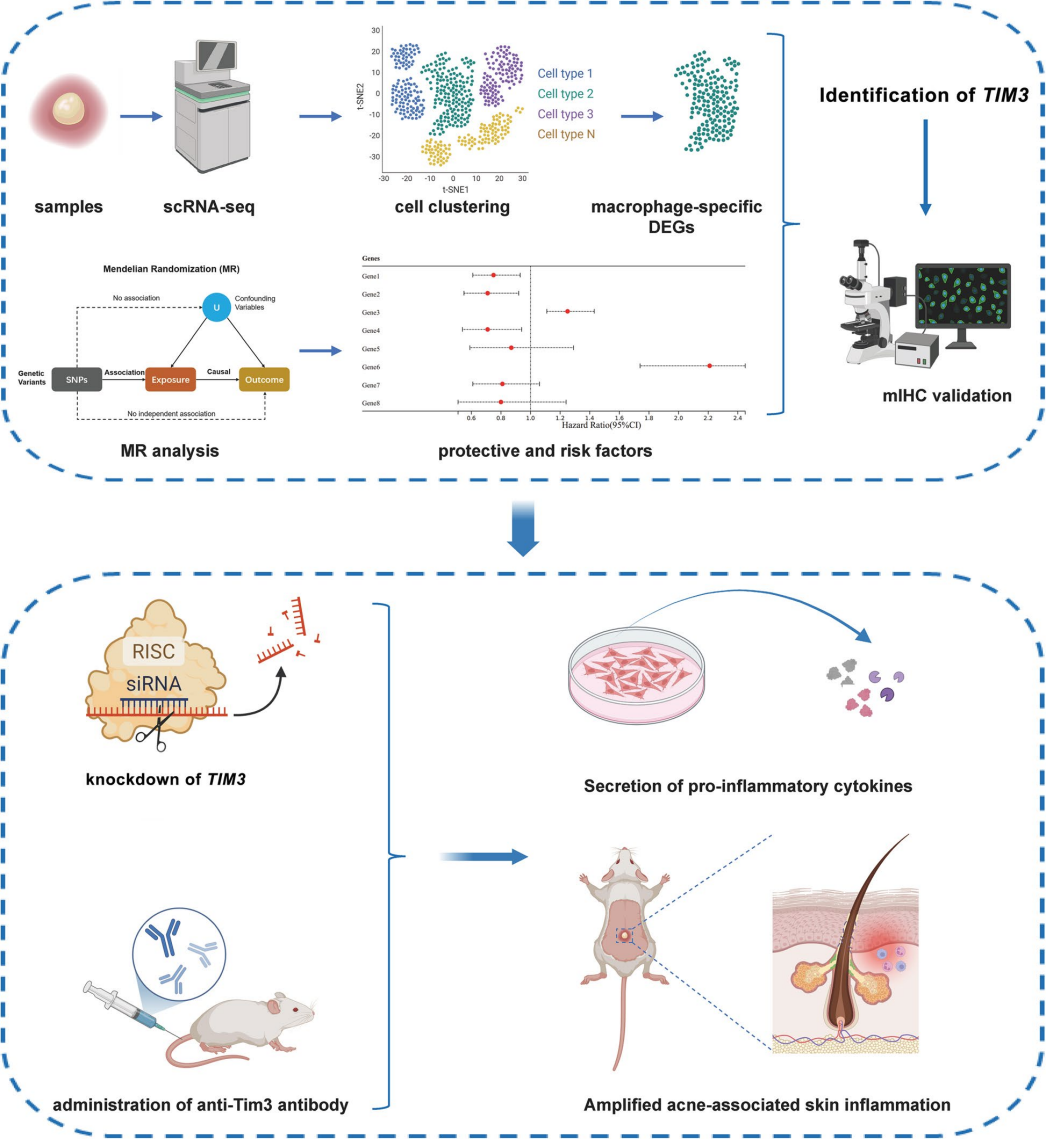
A brief description of the study design. A combined scRNA analysis was performed using SA, acne and normal samples. Then the data were normalized, scaled, batch corrected, dimensional reduced, and clustered. Macrophage-specific DEGs were screened and were taken intersection with MR screened plasma proteins. *TIM3* was identified as the key gene and the expression of TIM3 was validated via mIHC. Furthermore, the function of *TIM3* was investigated through knockdown of *TIM3* in HaCaT cells and administration of anti-Tim3 antibody in mice model, revealing that knockdown or blockage of TIM3 could amplify the SA associated inflammation. Some elements in Fig. 1 were created with BioRender.com

The study design is summarized in Fig. 1, and the detailed workflow is illustrated in Fig. S1. This investigation was divided into two complementary phases: the initial identification of *TIM3* via in silico approaches, followed by comprehensive functional exploration through in vitro and in vivo experimental models. Specifically, scRNA-seq data were collected from 9 samples at West China Hospital, comprising 6 from SA samples and 3 from normal skin samples. Concurrently, data from 6 acne lesions and 6 normal skin samples were retrieved from the Gene Expression Omnibus (GEO) database (GSE175817) to identify macrophage-specific DEGs. Integrating these macrophage-specific DEGs with MR analysis, *TIM3* was prioritized as a key gene implicated in SA development. The expression of TIM3 protein was subsequently validated using mIHC staining. To further investigate the functional role of TIM3 in SA pathogenesis, *TIM3* was knocked down using small interfering RNA (siRNA) in in vitro models, while an anti-Tim3 antibody was administered to block Tim3 signaling in in vivo models (Fig. 1). Detailed clinical characteristics and representative images of the SA patients are provided in Fig. S2.

### Combined analysis of scRNA data of SA, acne and normal samples

**Fig. 2:**
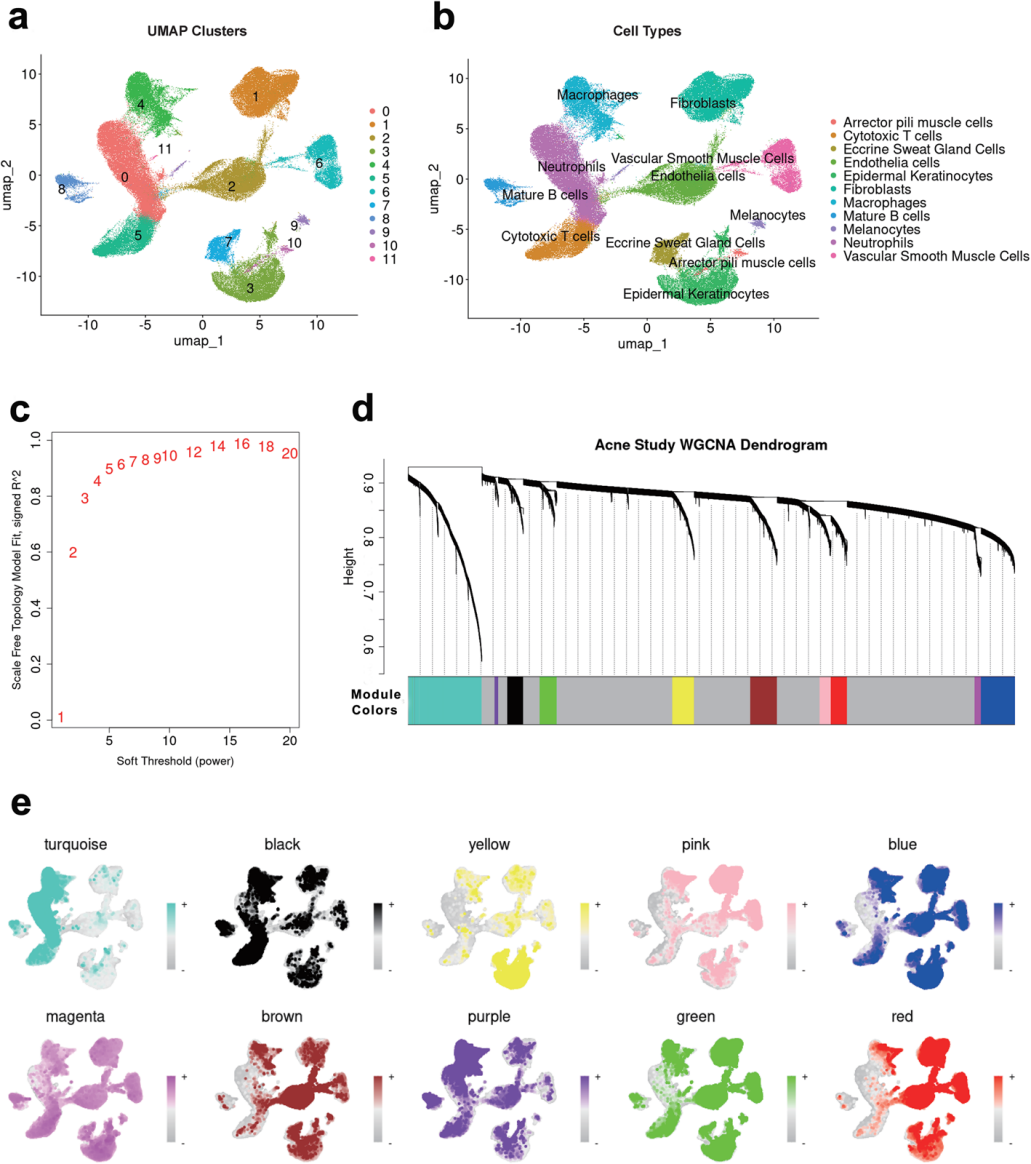
Macrophages serve as the important cell clusters in SA development. **a**-**b** UMAP before and after manual annotation. A total of 12 clusters were calculated finally 11 cell clusters were annotated and they were neutrophils, fibroblasts, endothelial cells, epidermal keratinocytes, macrophages, cytotoxic T cells, vascular smooth muscle cells, eccrine sweat gland cells, mature B cells, melanocytes, and arrector pili muscle cells. **c** The soft-threshold for scRNA WGCNA analysis was selected as β = 6 with the criteria of R^2^ > 0.9 for scale-free topology. **d** A total of 11 modules (grey module included) are detected as shown by the dendrogram in scRNA WGCNA analysis. **e** The gene activities are shown in UMAP. Deeper colors indicate higher gene activities. All gene modules showed high gene activities for macrophages

To obtain a comprehensive landscape of cellular heterogeneity and distribution during SA development, a joint analysis of scRNA-seq data was performed on the aforementioned 21 samples: 6 SA samples, 6 acne samples, and 9 normal samples. Prior to analysis, cells were filtered using stringent quality control criteria, including the threshold of genes more than 200 and less than 2500, and mitochondrial genes below 5% (Fig. S3a-b). The expression profiles of genes contributing to the first two principal components are visualized in a heatmap (Fig. S3c). Batch effects were effectively mitigated through the application of the “harmony” algorithm (Fig. S3d). With a resolution of 0.1, a total of 12 cell clusters were detected by uniform manifold approximation and projection (UMAP) (Fig. 2a). The top 5 marker genes were subsequently visualized and utilized for manual annotation (Fig. S3e), resulting in the identification of 11 annotated cell types (Fig. 2b); the heatmap shows the expression profiles of these marker genes (Fig. S3f). Visualization of cell clusters for each group revealed that macrophages and neutrophils, identified as myeloid cell populations, were notably enriched in both SA and acne groups (Fig. S3g-i).

To screen important cell clusters involved in SA development, the single-cell WGCNA was conducted. The soft threshold for network construction was set as β = 6 (Fig. 2c), leading to the detection of 11 distinct co-expression modules (Fig. 2d; genes not belonging to any other modules were allocated to the gray module). Module eigengene activities were calculated, revealing high activity predominantly in macrophages across all modules (Fig. 2e), underscoring their critical role in SA development. Although neutrophils were enriched in SA group, their limited representation constrained further validation; thus, macrophages were prioritized for downstream analysis.

On the basis of the expression profile of macrophage populations, DEG analysis revealed a substantial number of differentially expressed genes, with 2745 commonly upregulated and 3210 common down-regulated macrophages-specific DEGs in 4 SA samples. Functional enrichment analysis of these DEGs indicated a strong association with immune-related biological processes and inflammation-related pathways (Fig. S4). In summary, our integrated scRNA analysis of SA, acne, and normal samples successfully identified 11 annotated cell clusters. Further exploration of macrophage-specific DEGs within these clusters facilitated the identification of key genes implicated in SA pathogenesis.

### *TIM3* was identified as a key gene in SA development

**Fig. 3:**
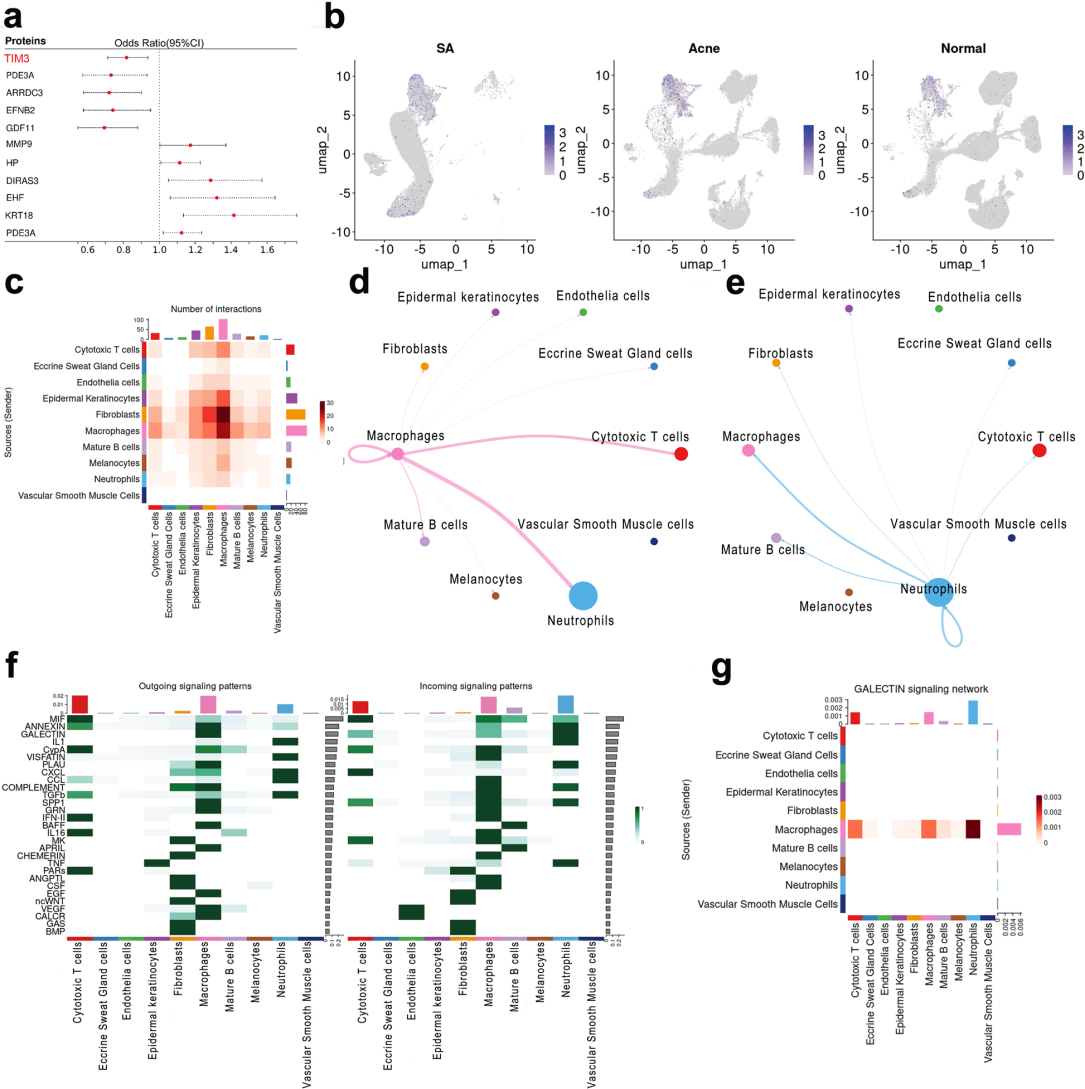
Identification of *TIM3* as the key gene in SA development. **a** Through combining the results of MR analysis and common DEGs, TIM3, PDE3A, ARRDC3, EFNB2, and GDF11 showed protective effects against SA development, while MMP9, HP, DIRAS3, EHF and KRT18 were risk factors in SA development. **b** In our scRNA analysis, *TIM3* was down regulated in macrophages in SA conditions compared with normal and acne group as the scatter plot showed. **c** The heatmap showed the number of interactions among 11 types of cells. **d**- **e** The cell–cell communication for macrophages and neutrophils. **f** The outgoing signaling patterns and incoming signaling patterns. **g** The cell–cell communication patterns of galectin signaling network as shown by heatmap

To identify key genes involved in SA development, an integrative approach combining MR analysis with scRNA-seq was employed. A comprehensive MR screening of 3,282 plasma proteins for associations was conducted, and 176 plasma proteins significantly associated with acne risk were found. Several candidate genes were identified (Fig. 3a) by intersecting these proteins with common DEGs from scRNA-seq and focusing on genes that were significant in at least two MR methods. Among these, significantly decreased odds ratios (ORs) across all four MR methods—inverse-variance weighted (IVW), weighted median, weighted mode, and MR-Egger—were demonstrated by TIM3, an immune checkpoint gene. A protective role in acne development is suggested for TIM3 by this consistent finding. The funnel plot revealed that the heterogeneity for TIM3 in MR analysis was acceptable (Fig. S5a), whereas the leave-one-out analysis revealed that the MR results for TIM3 were stable (Fig. S5b). Consistent with the MR findings, downregulation of *TIM3* expression in macrophages from SA group compared to controls was revealed by scRNA-seq data (Fig. 3b and Fig. S5c-d).

To elucidate TIM3-related intercellular communication networks potentially involved in SA pathogenesis, CellChat analysis was performed (Fig. 3c–e). The heatmap summarized outgoing and incoming signaling patterns across known pathways, suggesting the involvement of the TIM3-related galectin pathway in SA (Fig. 3f). Notably, an increase in the predicted interaction strength of galectin signaling between macrophages and other cells in SA tissues was also observed (Fig. 3g). Given that GAL9 is a principal ligand of TIM3 [22], the corresponding cell–cell communication patterns and GAL9-related pathways are shown in Fig. S6 and Fig. S7, respectively.

**Fig. 4:**
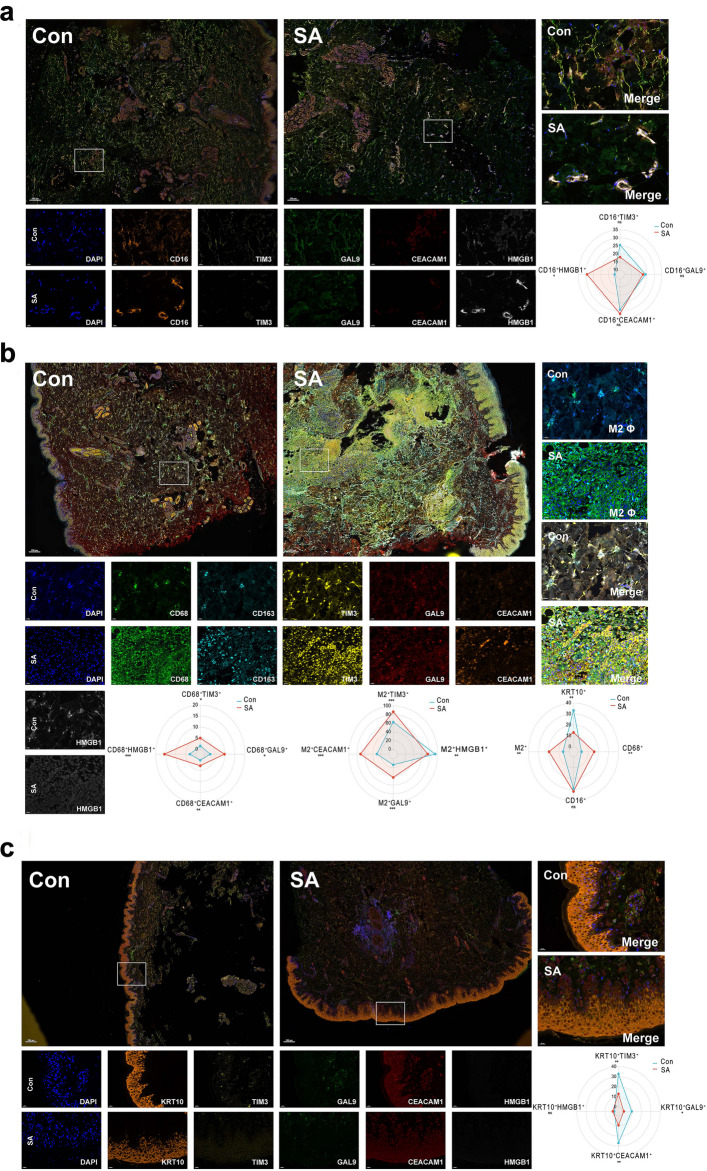
The expression of TIM3 and its ligands in neutrophils, keratinocytes and macrophages shown by mIHC. The quantitative analysis is shown in radar plot. **a** The expression profile of TIM3 and its ligands in neutrophils. TIM3 shows no significance in neutrophils between SA and normal skin tissues. **b** The expression profile of TIM3 and its ligands in macrophages. In all macrophages, TIM3 is up-regulated in SA condition, which may be due to the up-regulation of TIM3 in M2 macrophages, suggesting the role of TIM3 in M2 polarization. The percentage for neutrophils, keratinocytes, total macrophages and M2 macrophages is also compared and shown in the radar plot. Among these cells, total macrophages and M2 macrophages were enriched in SA tissues, keratinocytes were enriched in normal tissues while neutrophils showed no significant differences between SA and normal tissues. **c** The expression profile of TIM3 and its ligands in keratinocytes between SA and normal skin tissues. TIM3 is significantly down-regulated in SA conditions suggesting its immune-checkpoint function may be compromised

Although TIM3 was originally found on Th cells and serves as a marker for T-cell exhaustion, recent studies have focused on its role in non-T cells, especially myeloid cells [22–24]. Therefore, the expression of TIM3 was investigated through mIHC on myeloid cells (neutrophils and macrophages). Fewer TIM3^+^ neutrophils in SA group compared to normal skin were revealed by mIHC (Fig. 4a). Interestingly, an increased number of TIM3^+^ macrophages in SA group was observed, which appeared to result specifically from enrichment of TIM3^+^ M2 macrophages (Fig. 4b). In addition, through mIHC, the expression of TIM3 in keratinocytes from normal skin and the reduced presence of TIM3^+^ keratinocytes in SA tissues were reported for the first time (Fig. 4c). Therefore, we inferred that TIM3 could function as an immune checkpoint not only in immune cells but also in keratinocytes during SA pathogenesis. By this integrated approach combining macrophage-specific DEGs, MR analysis, and mIHC staining, *TIM3* was identified as a key gene involved in the development of SA.

### Knockdown of *TIM3 *enhances the secretion of pro-inflammatory cytokines induced by *P. acnes* in HaCaT cells

**Fig. 5:**
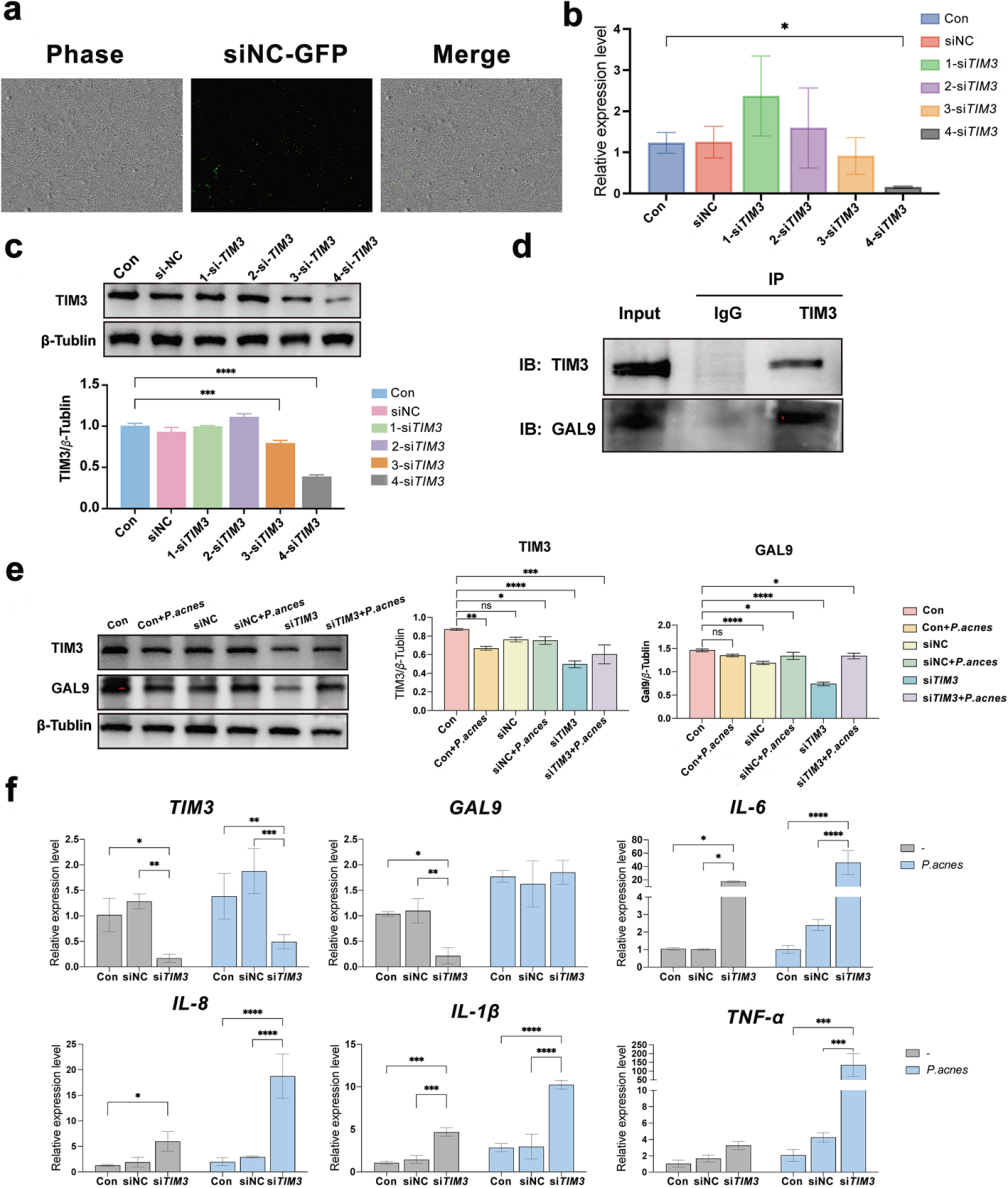
Knockdown of *TIM3* enhances the secretion of pro-inflammatory cytokines induced by *P. acnes* in HaCaT cells. Effects of *TIM3* knockdown on protein expression and inflammatory cytokines in HaCaT cells. **a** Representative image of HaCaT cells transfected with siNC‑GFP (bar = 100 µm). **b** Silencing efficiency of si*TIM3* at the mRNA level in HaCaT cells determined by qRT‑PCR. **c** Knockdown efficiency of si*TIM3* at the protein level in HaCaT cells assessed by western blot. **d** Co‑IP confirmed the physical interaction between TIM3 and GAL9 in HaCaT cells following *TIM3* knockdown. **e** Changes in TIM3 and GAL9 protein expression before and after *P. acnes* stimulation. **f** Expression changes of *TIM3, GAL9, IL‑6, IL‑8, IL‑1β* and *TNF‑α* before and after *P. acnes* stimulation. Data are presented as mean ± SD. *P < 0.05

Because *TIM3* may serve as an immune checkpoint in SA development, its function in keratinocytes was explored through knockdown approaches. First, HaCaT cells were transfected with siNC-GFP, and the transfection efficiency exceeded 60% at 6 h posttransfection (Fig. 5a). To assess the effect of *TIM3* knockdown in HaCaT cells, four si*TIM3* candidates were designed and screened by qRT‑PCR. At 24 h posttransfection, *TIM3* mRNA levels were significantly lower in the 4-si*TIM3* group (*P* < 0.05) (Fig. 5b), and *TIM3* protein expression levels were markedly lower at 72 h (*P* < 0.001) (Fig. 5c). Therefore, 4-si*TIM3* was used for subsequent experiments.

The results of the CellChat analysis described above revealed that the galectin pathway might play a role in SA development. Although GAL9 has been reported to be a ligand for TIM3 in lymphocytes [25], their interaction in HaCaT cells has not been established. Co-IP revealed that GAL9 also interacts with TIM3 in HaCaT cells (Fig. 5d). *TIM3* was knocked down in HaCaT cells with heat-inactivated *P. acnes*. At 72 h after stimulation, the expression of TIM3 and its ligand GAL9 was significantly reduced (Fig. 5e). At 48 h poststimulation, in the control group, the mRNA expression of *IL-6*, *IL-8*, and *IL-1β* was upregulated, whereas no significant differences in *TNF-α* mRNA expression were observed, and these increases were further augmented in *TIM3*-knockdown cells compared with negative controls (Fig. 5f). These findings showed that knocking down *TIM3* in keratinocytes could amplify SA-related inflammation, suggesting that TIM3 plays a role as an immune checkpoint in keratinocytes.

### Administration of anti-Tim3 exacerbates acne and inflammation in a mouse model

**Fig. 6:**
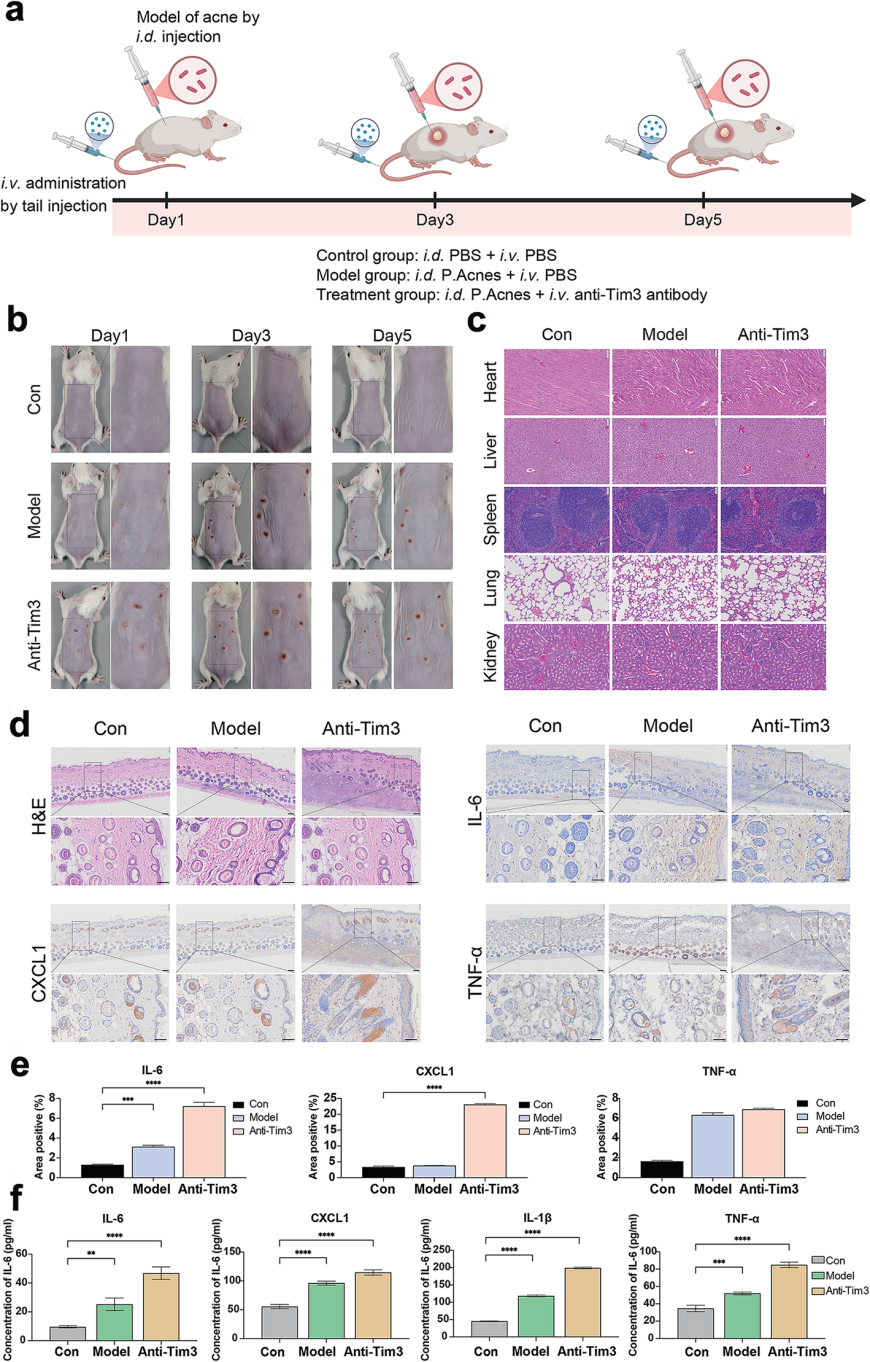
Anti-Tim3 antibody amplifies *P. acnes*–induced inflammation in a mouse acne model. **a** A brief scheme of the in vivo experiments (*i.d.* = intradermal, *i.v.* = intravenous). Some of the elements were created in BioRender.com. **b** Representative images of skin surface changes in the dorsal area of mice. **c** H&E staining of heart, liver, spleen, lung, kidney to assess systemic involvement. **d** H&E analysis of skin lesions and IHC staining for IL-6, CXCL1, and TNF-α in skin tissues (top, scale bar = 200 µm, bottom, scale bar = 50 µm). **e** Quantitative analysis of cytokine expression (IL-6, CXCL1, TNF-α) from IHC images using ImageJ software. **f** Serum cytokine levels by ELISA. *P < 0.05, **P < 0.01, ***P < 0.001, ****P < 0.0001

To evaluate the influence of Tim3 on *P. acnes* infection in vivo, an acne model was established in BALB/c mice by intradermal injection of *P. acnes*, followed by intravenous administration of either a Tim3–blocking antibody or a vehicle control. A brief scheme is shown in Fig. 6a. Acne lesions were monitored and analyzed on Days 1, 3, and 5 posttreatments (Fig. 6b). The findings revealed successful lesion formation (1–5 mm) on Day 1 in both the model and anti-Tim3 groups. By Day 3, lesions persisted and were enlarged in the anti-Tim3 group, whereas the lesions in the model group had a partial regression by Day 5; however, compared with those in the model group, the lesions in the anti-Tim3 group remained more severe.

Histological assessment of the visceral organs through H&E staining revealed no significant inflammatory changes across the groups (Fig. 6c), suggesting that the inflammatory effects were primarily localized to the skin. Histological examination of skin sections (Fig. 6d) revealed normal epidermal architecture in the control group with minimal dermal inflammatory cells. The model group exhibited epidermal thickening and substantial dermal inflammatory infiltration with enlarged hair follicles. Compared with the model group, the anti-Tim3 group demonstrated markedly enhanced inflammation characterized by pronounced epidermal hyperplasia, extensive dermal infiltration, and severely distended hair follicles with evidence of rupture.

To further investigate the molecular basis of the heightened inflammation, immunohistochemistry (IHC) staining for the inflammatory cytokines IL-6, CXCL1, and TNF-α was performed on skin sections (Fig. 6d). IHC staining revealed upregulated expression of IL-6, CXCL1, and TNF-α in the anti-Tim3 group compared with the control group, indicating increased local proinflammatory signaling (Fig. 6e). Systemic cytokine analysis via ELISA (Fig. 6f) confirmed heightened serum levels of IL-6, CXCL1, IL-1β, and TNF-α in both *P. acnes*-exposed groups, with markedly greater increases in IL-6 and TNF-α in the anti-Tim3 group than in the model group.

These results demonstrate that anti-Tim3 antibody administration significantly exacerbates the inflammatory response in *P. acnes*-induced acne, as evidenced by increased lesion severity, more extensive histological inflammation, increased local proinflammatory cytokine expression, and elevated systemic inflammatory marker expression. These findings suggest that TIM3 plays a critical immunoregulatory role during *P. acnes* infection, with its inhibition resulting in an aggravated disease phenotype.

## Discussion

In this study, scRNA and MR analyses converged on TIM3 as a key mediator of SA development at the transcriptome level. Subsequent mIHC confirmed its protein expression in myeloid cells (macrophages and neutrophils) and keratinocytes, and functional experiments in vitro and in vivo indicated that TIM3 acts as an immune checkpoint in SA.

**Fig. 7:**
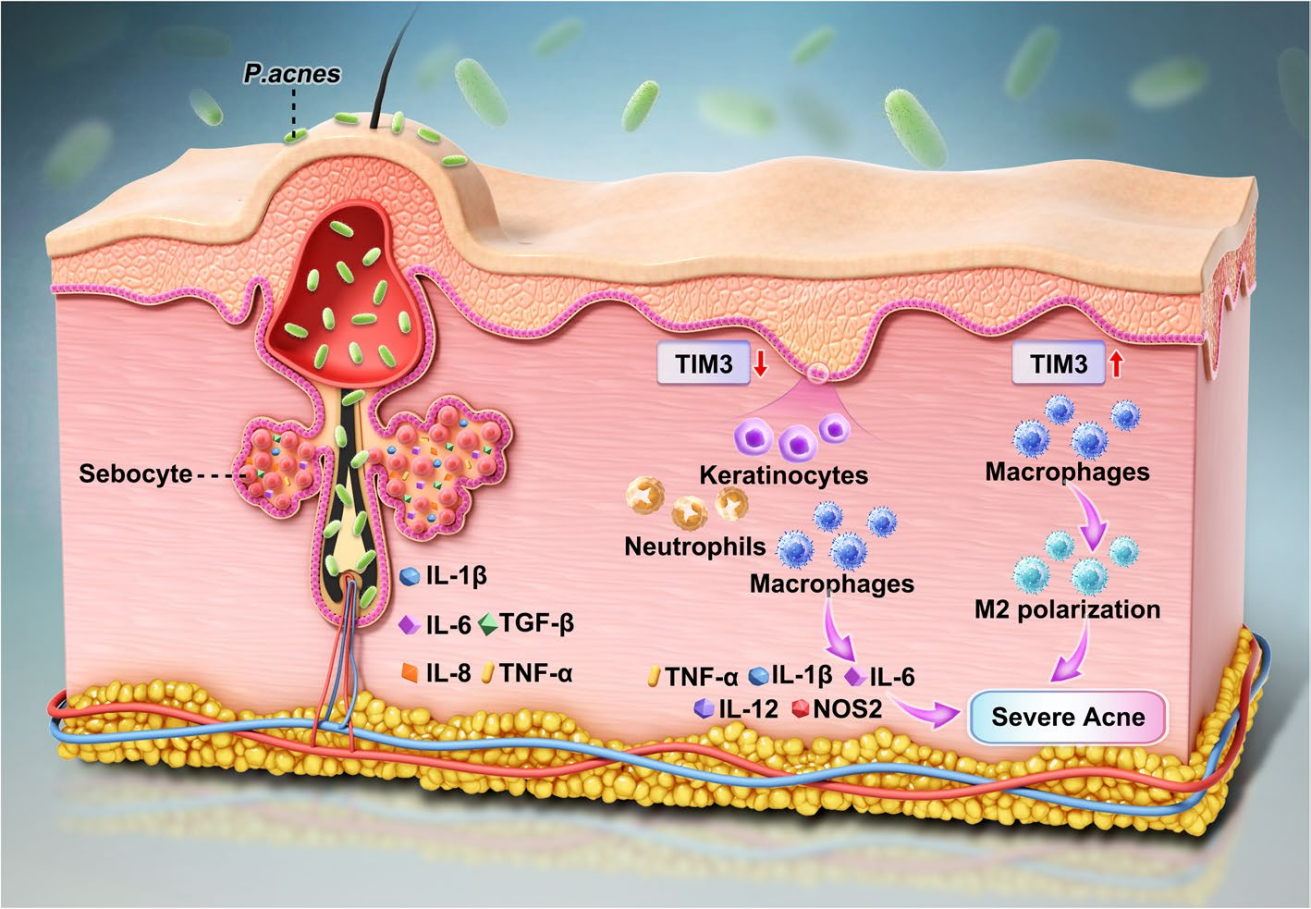
The proposed mechanisms of TIM3-mediated immunomodulation in SA development

In macrophages, TIM3 primarily exerts a net anti-inflammatory effect. Macrophage TIM3 can inhibit the expression of proinflammatory cytokines such as TNFα, IL-1β, IL-6, IL-12, and NOS2 in dextran sodium sulfate (DSS)-induced colitis [26]. Similarly, in *Listeria monocytogenes* infection, TIM3 expression in macrophages limits the phagocytosis of bacteria through interactions with the transcription factor nuclear factor NRF2 and the down-regulation of CD36 and the heme oxygenase 1-IL1β pathway [27]. In acute sepsis, TIM3 is up-regulated in peripheral blood mononuclear cells, whereas TIM3 expression is suppressed in severe sepsis [18]. In addition, TIM3 plays an important role in macrophage polarization. Macrophage TIM3 could serve as an adaptor for STAT1 through its Tyr256 and Tyr263 residues at the intracellular tail. As a result, the expression of the microRNA miR-155 is suppressed, and SOCS1 activity is increased, leading to the repression of JAK signaling and ultimately the promotion of M2 macrophage polarization [28]. Through mIHC, our study revealed that TIM3 expression is increased in macrophages under SA conditions. Further investigation revealed that TIM3 is up-regulated in M2 macrophages in SA patients, as revealed by mIHC. Previous studies have reported that TIM3 can promote M2 macrophage polarization, the level of TIM3 in macrophages increases in response to pathogenic factors, and TIM3 functions mainly as a regulator of macrophage polarization to suppress inflammation in SA tissues (See Fig. 7). However, for neutrophils, TIM3 expression tended to decrease slightly in SA tissues, and no significant differences were detected. The limited neutrophil infiltration in our collected SA tissue may account for these findings.

TIM3 also plays important roles in inflammatory skin diseases such as psoriasis and atopic dermatitis. In psoriasis patients, Th1/Th17 effectors fail to appropriately up-induce TIM3, and TIM3–negative cells produce more IFN-γ/IL-17, consistent with loss of a local “brake” on type-1/17 immunity [29]. Pharmacological restoration of the axis also has a proof-of-concept: a stable form of galectin-9 (a TIM3 ligand) suppresses contact hypersensitivity and IL-23–driven psoriasiform dermatitis, reducing IL-17/IL-22 and epidermal STAT3 activation [30]. In atopic dermatitis, the TIM3 checkpoint and its ligand GAL9 form a disease-relevant axis that shapes both adaptive and epithelial inflammation. Patient studies have shown elevated serum GAL9 levels, increased frequencies of TIM3⁺ CD4 T cells, and positive correlations with clinical severity indices (SCORAD/EASI), IgE and eosinophils; moreover, GAL9 engagement limits Th1/Th17 proliferation/survival while promoting Th2/Th22 polarization, which is characteristic of AD [31]. However, few studies have directly reported the role of TIM3 in keratinocytes.

Interestingly, the expression of TIM3 on keratinocytes in normal skin tissues was also detected, which has not been reported in other studies. Keratinocytes are the main cellular components of the epidermis and help maintain physical barriers [32–35]. In addition to their barrier function, keratinocytes can also serve as regulators of skin immunity and inflammation by responding to as well as producing chemokines, cytokines and growth factors and play crucial roles in inflammatory skin diseases such as psoriasis, atopic dermatitis, and cutaneous lupus erythematosus [36–39]. Various cytokine receptors and pathogen-associated molecular pattern (PAMP) receptors expressed on keratinocytes, such as Toll-like receptors (TLRs), RIG-I-like receptors (RLRs), nucleotide-binding oligomerization domain-like receptors (NLRs), and C-type lectin receptors (CLRs), are important for immune and inflammation regulatory functions [40–43]. TIM3 has been reported to inhibit TLR2/4/NF-κB signaling in myeloid cells, therefore [17, 44], whether TIM3 could be expressed on keratinocytes and serve as an immune regulator was of interest. Through mIHC, TIM3 was found to express in keratinocytes of normal skin tissues and down-regulated in keratinocytes of SA tissues. In addition, in vitro and in vivo experiment found that knock-down or blockage of TIM3 could amplify the SA associated inflammation, which first indicates that TIM3 not only functions in immune cells but also serves as an immune checkpoint in keratinocytes.

Acne lesions are fueled by *P. acnes* triggered innate and adaptive immnue responses. During acne pathogenesis, sebocytes and monocytes/macrophages can activate NLRP3 inflammasomes and release IL-1β [20, 45, 46]. In keratinocytes, TIM3 signaling may serve as an immune checkpoint to limit NF-κB “priming” and NLRP3 activation. Therefore, the down-regulation of TIM3 might lead to the disinhibition of NF-κB and NLRP3 related pathways, amplifying the keratinocyte-mediated inflammation, and ultimately promoting SA pathogenesis.

Our study has several limitations. First, although CellChat-based analyses suggest a role for TIM3-related intercellular signaling, the underlying molecular mechanisms driving the role of TIM3 in SA remain to be defined, and dedicated in vitro and in vivo experiments are needed. Second, several conclusions rely on computational inference (e.g., pathway and ligand–receptor interactions predicted by CellChat), which should be interpreted as hypothesis-generating and warrant experimental validation. Third, we combined scRNA-seq data from SA samples with publicly available datasets; despite the use of shared 10 × Genomics/Illumina platforms and batch-correction procedures, residual bias may persist. Future work should include contemporaneously profiled SA, acne, and normal samples, ideally using matched scRNA-seq and/or spatial transcriptomics, to minimize batch effects.

In summary, TIM3 was identified as a crucial player during SA. TIM3 is expressed in keratinocytes in normal skin but is downregulated in SA, and its knockdown or blockade exacerbates SA-associated inflammation, indicating an immune checkpoint role for TIM3 in nonimmune cells. On the basis of the results of the scRNA and MR analyses, we identified TIM3 as a pivotal checkpoint in SA development. Mechanistically, in macrophages, TIM3 appears to promote M2 polarization within SA tissues, potentially dampening inflammation; in neutrophils, TIM3 expression tends to decrease, which may increase the production of proinflammatory cytokines. Together, these cell type–specific effects converge to amplify inflammatory milieu when TIM3 signaling is reduced. Notably, these findings broaden the canonical view of TIM3 beyond its role in hematopoietic compartments. Our results highlight a tractable avenue for therapeutic modulation in SA and point to keratinocyte-centered immune checkpoint mechanisms as a promising direction for elucidating the pathogenesis of inflammatory skin diseases.

## Methods & materials

### Sample collection and single-cell suspension preparation

A total of 6 SA samples and 3 normal samples were collected from West China hospital for scRNA-seq. Both SA and normal samples were obtained from 4 mm punch biopsies on the back. Clinical information was collected from medical records (Fig. S2a-b). The SA pustules were digested in an enzyme solution (Millipore, USA) for 1 h at 37 °C. Following digestion, the cells were harvested and centrifuged at a maximum speed of 400 rcf. The supernatant was gently removed, leaving behind the cell pellet, which was subsequently resuspended in 1 mL of 1X PBS containing 0.04% BSA. This washing process was repeated twice. Afterward, an appropriate amount of PBS was added to the cell pellet to create a single-cell suspension with a concentration nearing the desired target. A wide-bore pipette tip was used during resuspension to reduce potential cell damage. Cell debris and clumps were eliminated via 30 µm MACS SmartStrainers and 40 µm Flowmi™ Cell Strainers. The cell concentrations were measured via an automated cytometer (Thermo Fisher Scientific; AMAQAF1000), and the sample volume was adjusted according to the optimal cell concentration recommended by 10 × Genomics and the target capture requirements. Once the suspension was ready, it was immediately chilled on ice for subsequent GEM generation and reverse transcription, which was conducted by Annoroad Gene Tech. (Beijing) Co., Ltd.

Library preparation was performed via the Chromium Next GEM Automated Chip G Single Cell Kit (10 × Genomics, Cat#PN-1000146) in accordance with the manufacturer’s instructions. Briefly, cells, partitioning oil, and beads were loaded onto a Chromium Chip G to produce single-cell GEMs. These GEMs were then collected and subjected to linear amplification in a C1000 Touch Thermal Cycler under the following conditions: 72 °C for 5 min, 98 °C for 30 s, followed by 12 cycles of 98 °C for 10 s, 63 °C for 30 s, and 72 °C for 1 min. The emulsions were disrupted using the Recovery Agent and cleaned with Dynabeads. Indexed sequencing libraries were subsequently constructed, purified, and sequenced on an Illumina NovaSeq platform at Annoroad Gene Tech. (Beijing) Co., Ltd.

### Standard single cell sequencing analysis

scRNA data for acne and normal tissues were obtained from the Gene Expression Omnibus (GEO) database (GSE175817). scRNA-seq analyses were performed on acne lesions and paired nonlesional tissues from each acne patient (6 patients), and a total of 12 samples were included in this dataset [47].

CellRanger software (v7.1.0) was used to demultiplex the Illumina BCL output into FASTQ files. Seurat (v5.0.3) was utilized to integrative analysis and comparative analysis workflows to perform all the scRNA-seq analyses [48]. For quality control and filtering out low-quality cells, only cells expressing more than 200 genes (defined as genes detected in at least 3 cells), fewer than 2500 genes, and fewer than 5% mitochondrial genes were selected. A total of 14,426 cells were included in further analysis.

Before cell clustering, scRNA-seq data for SA patients, normal samples collected, and the scRNA-seq data in GSE175817 were integrated and batch corrected via the “harmony” (v0.1.1) method. Then the shared nearest-neighbor graph and identified clusters in the network were computed via the Louvain algorithm with a resolution of 0.1. The uniform manifold approximation and projection (UMAP) method was used for visualization of unsupervised clustering. Differential gene expression or marker gene expression was determined by the “FindAllMarkers” function with the default Wilcoxon’s rank-sum test either as one versus the rest or as a direct comparison with parameters FDR < 0.05 and | log_2_(fold change) |> 0.25. All the cell clusters were manually annotated on the basis of the top 5 markers.

### Important cell cluster selection and DEG analysis

DEG analysis was performed on the basis of the gene expression profiles of the macrophages. Using the “FindMarkers” function, DEGs between SA and normal samples and between SA and acne samples were screened. The threshold for screening DEGs is FDR < 0.05 and | log_2_(fold change) |> 0.25. The intersections of these two sets of DEGs and were identified as common DEGs involved in SA development. Gene Ontology (GO) and Kyoto Encyclopedia of Genes and Genomes (KEGG) functional enrichment analyses were performed for these common DEGs.

### Two-sample MR study of the association between plasma protein levels and the risk of acne

A series of two-sample MR studies were performed on the associations between plasma protein levels and the risk of acne using summary-level genetic data retrieved from the MRC Integrative Epidemiology Unit (IEU) Open GWAS database (https://gwas.mrcieu.ac.uk/). The GWAS summary data on protein levels were described by Sun et al. [49], and the GWAS summary data of outcomes were from the FinnGen cohort (https://www.finngen.fi/en/cohort_and_data). The odds ratio (OR) of the risk of acne associated with the expression of plasma proteins were obtained via inverse-variance weighted (IVW) meta-analysis with a multiplicative random-effects model. Three sensitivity analyses (i.e., weighted median, MR‒Egger and MR pleiotropy residual sum and outlier (MR-PRESSO) methods) were conducted to examine the presence of horizontal pleiotropy and address the detected heterogeneity. The heterogeneity of SNP effects was also examined via Cochran’s test.

### Identification of TIM3 as a crucial gene in SA development

The intersections between the plasma proteins showing a significant impact on SA development (mainly via the IVW method) and the common DEGs were taken. TIM3 was found to be significant through IVW, weighted median, weighted mode and MR‒Egger methods in MR analysis and was identified as a crucial gene. A dot plot and scatter plot (UMAP) were used to show the expression profiles of TIM3.

The “CellChat” R package was used to conduct cell‒cell interaction analysis, which is based on the known interactions between receptors and ligands and cofactors [50]. The galectin signaling pathway and visualized it in detail, since it involves GAL9-TIM3 signaling.

### Multiplex immunohistochemistry (mIHC)

To validate the expression status of TIM3 and its protein ligands in myeloid cells (neutrophils and macrophages in this study), the multi-color fluorescent immunohistochemical staining kit (Absin, Cat#50,015) was applied to perform polychromatic immunofluorescence staining, which is based on the tyramide signal amplification (TSA) technique. Since keratinocytes also play important roles in skin inflammation, the expression of these target proteins in keratinocytes was also validated.

For our staining, SA lesions derived from patients and normal skin tissues from one volunteer with pigmented nevi were fixed via 4% paraformaldehyde and then embedded in paraffin wax. Before staining, these 5 μm tissue sections were dewaxed with xylene, cleared in 100% ethanol, and rehydrated through graded ethanols to water. The primary antibodies used for mIHC staining were anti-KRT10 (HUABIO, Cat#ET1609-75), anti-CD16 (HUABIO, Cat#HA721149), anti-CD68 (Abcam, Cat#ab955), anti-CD163 (HUABIO, Cat#ET1704-43), anti-TIM3 (Abcam, Cat#ab241332), anti-GAL9 (Abcam, Cat#ab69630), anti-HMGB1 (Proteintech, Cat#82,973–1-RR), and anti-CEACAM1 (Abcam, Cat#ab300061) antibodies. The tissue sections were subjected to microwave-induced antigen retrieval, and the retrieval buffer used was either Tris–EDTA buffer (pH = 9.0) or sodium citrate buffer (pH = 6.0) according to the antibody manual. Then, the 0.3% hydrogen peroxide was used to block endogenous peroxidase activity in the tissue sections. The tissue sections were washed with PBST and blocked with 5% serum for secondary antibodies (goat) for 20 min. To achieve optimal staining effects, the primary antibodies were incubated with the tissue sections overnight at 4 °C. After incubation with primary antibodies, the tissue sections were incubated with secondary antibodies conjugated with horseradish peroxidase (HRP) for 10 min at 37 °C, followed by incubation with TSA fluorescence dye for 10 min at 37 °C. For multiple staining, the antigen retrieval step was started on the sections to remove the previous binding antibodies, and this step was repeated until all the targets were stained. Finally, the tissue sections were stained with DAPI for 5 min at room temperature and mounted with antifade fluorescence mounting medium (Abcam, Cat#ab104135). The stained tissue sections were scanned via the Vectra Polaris Automated Quantitative Pathology Imaging System (Akoya Biosciences, Inc., Marlborough, MA). The scanned files were visualized through PhenoChart (v1.1.0) and analyzed through inForm (v2.4.2) software.

### Cell culture and treatment

The human keratinocyte cell line (HaCaT cell) was obtained from our laboratory and authenticated by short tandem repeat (STR) analysis. The cells were cultured in MEM medium (Gibco) supplemented with 10% fetal bovine serum (FBS, Viva Cell, Shanghai,China), and maintained at 37˚C under a humidified atmosphere of 5% CO2.

### Cell transfection

The* TIM3* siRNA (sense(5'−3') GGUAUUCUCAUAGCAAAGATT, antisense(5'−3') UCUUUGCUAUGAGAAUACCTT) and scramble siRNA (as negative control) were synthesized by GenePharma (Shanghai, China) and transfected using CALNP™ RNAi in vitro (D-Nano Therapeutics, Beijing, China). HaCaT cells were seeded in 6-well plates and allowed to reach 70%–80% confluence prior to transfection. Transfection was carried out for 24 h before harvest for quantitative real-time PCR, and 72 h for Western blotting analysis.

### Bacterial culture

*Propionibacterium acnes* (*P. acnes*, ATCC6919) was obtained from the China General Microbiological Culture Collection Center (CGMCC, Beijing). Bacteria were inoculated into brain–heart infusion (BHI) broth and incubated anaerobically at 37 °C for 48 h. Bacterial cells were collected by centrifugation, washed twice with PBS, and subsequently resuspended in PBS. The optical density at 600 nm (OD600) was measured using a spectrophotometer to standardize the bacterial suspension to a final concentration of 1 × 10^6^ CFU/mL for subsequent use.

### Mouse acne model and drug administration

Seven-week-old male BALB/c mice were subjected to depilation of the dorsal skin. Subsequently, a skin abscess model was established by intradermal injection of 40 µL of *P. acnes* suspension containing 1 × 10^6^ CFU into the dorsal skin, once daily for five consecutive days. Animals were divided into the following groups and treated accordingly: Control group: Intradermal injection of 40 µL PBS in the same dorsal region and intravenous injection of 200 µL PBS into the tail vein, once daily for five days. Model group: Intradermal injection of 40 µL *P. acnes* suspension, daily for five days. Treatment group: Received intradermal injection of 40 µL *P. acnes* suspension and intravenous tail vein injection of 200 µL anti-Tim3 solution at 1.5 mg/mL, once daily for five days.

### Quantitative real-time PCR

As described previously [51], total RNA was extracted from HaCaT cell cultures using the Super Fast Pure Cell RNA Isolation Kit (Vazyme, Nanjing, China). RNA concentrations were determined, and 1 μg of total RNA was reverse-transcribed to cDNA using the HiScript III RT SuperMix for qPCR (+ gDNA wiper) kit (Vazyme, Nanjing China). Quantitative real-time PCR was subsequently performed with Chama SYBR qPCR Master Mix (Vazyme, Nanjing, China) on an ABI QuantStudio6 FLEX Q6 (ThermoFisher Scientific, Shanghai, China) according to the manufacturer’s instructions. The primers used for qPCR were listed in Table S1. Relative gene expression levels for *TIM3, GAL9, IL6, IL8, IL1β* and *TNFα* were normalized to *GAPDH* and analyzed using the 2^−∆∆CT^ method.

### Western blot analysis

After 72 h post-transfection, the transfected cells were washed with ice-cold PBS and lysed in RIPA buffer (Beyotime, Shanghai, China). Total cellular protein was harvested, and the protein concentration was determined by the bicinchoninic acid (BCA) assay (ThermoFisher Scientific, Shanghai, China). Equal amounts of protein (40 μg) were resolved on a 12.5% sodium dodecyl sulfate–polyacrylamide gel (SDS-PAGE, Epizyme Biotech, Shanghai, China) electrophoresis and subsequently transferred onto polyvinylidene difluoride (PVDF) membranes (Millipore, Billerica, MA). Membranes were then blocked with 5% (w/v) non-fat dry milk in TBST for 1.5 h at room temperature, followed by overnight incubation at 4 °C with primary antibodies against Tim3 (Catalog number: PQA1998M, Abmart, Shanghai, China) and Gal9 (Catalog number: ab153673, abcam, Shanghai, China) as well as β-Tublin (Catalog number: 10068–1-AP, proteintech,Wuhan,China). The next day, HRP-conjugated goat anti-mouse or anti-rabbit IgG secondary antibody (Catalog number: SA00001-1 and SA00001-2, 1:10,000, proteintech, Wuhan, China) were applied for 1 h at room temperature. After incubation, membranes were washed 3 times with TBST. Finally, the protein bands were developed using an enhanced chemiluminescence (ECL) substrate (abbkine, Wuhan, China) and visualized with an ECL detection system (Bio-Rad, Shanghai, China). Quantitative analysis of the detected bands was performed by ImageJ software (National Institutes of Health, USA).

### Co-immunoprecipitation experiments (Co-IP) assay

For Co-IP analysis, HaCaT cells were lysed in immunoprecipitation lysis buffer (Beyotime, Shanghai, China) supplemented with a 1% protease inhibitor cocktail (Beyotime, Shanghai, China). Cell lysates were incubated overnight with 10 µg of anti-TIM3 antibody (Catalog number: ab241332, abcam, Shanghai, China) at 4 °C on a rotator. The following day, lysates were incubated with 50 µL protein A/G magnetic beads (MCE, Shanghai, China) for 1 h at room temperature. The beads, with bound immune complexes, were then isolated using a magnetic rack to obtain the precipitate. The immunoprecipitates were washed five times with TBST. Finally, precipitated proteins were denatured in 5 × SDS loading buffer for 5–10 min and subsequently analyzed via western blotting.

### ELISA

ELISA kits for mouse IL-6 (EK206), CXCL1/KC (EK296), IL-1β (EK201B), and TNF-α (EK282) were purchased from Multi Sciences Biotech, Co. LTD. Blood samples were collected from mice prior to sacrifice. Serum was obtained by centrifugation, and ELISA experiments were performed according to the manufacturer’s instructions.

### Hematoxylin and eosin (H&E) staining

Skin specimens were fixed in 10% neutral buffered formalin for 24 h, dehydrated, and embedded in paraffin. Serial Sects. (3 μm) were cut and stained with hematoxylin and eosin according to standard protocols.

### Immunohistochemistry for mice skin section

Till the incubation of secondary antibody, the experimental procedure is the same as mIHC. And the primary antibodies used in this section are antibodies against IL-6, TNF-α, and CXCL1. After washing, slides were incubated with a goat anti-rabbit IgG secondary antibody (1:200) at 37 °C for 30 min. Signal was developed with DAB chromogen, and sections were counterstained with hematoxylin. Whole-slide bright-field images were acquired using a VS200 slide scanner and analyzed with OlyVIA v4.1 software.

### Statistical analysis

All the bioinformatic analyses were performed in R 4.5.1. All experiments were conducted with three independent biological replicates. Quantitative data are presented as mean ± standard deviation (SD). Statistical significance was determined using one-way ANOVA (for single-variable comparisons) or two-way ANOVA (for multifactorial analyses). Data processing, statistical analyses, and graphical representations were performed using GraphPad Prism 10 (GraphPad Software, La Jolla, CA). A *P* value ≤ 0.05 was considered statistically significant; P values > 0.05 were considered not significant.

## Supplementary Information

Supplementary Material 1: Fig. S1. Workflow of the whole study. Fig. S2. Clinical data of SA patients. (a) Clinical data of patients involved in scRNA-seq. (b) Clinical data of patients involved in mIHC. (c)Representative images of SA patients. Fig. S3. scRNA data processing. (a) Violin plot before quality control. (b) Violin plot after filtering cells with the threshold of genes more than 200 and less than 2500, and mitochondrial genes below 5%. (c) Heatmap for the expression profiles of the first 2 principal components. (d) UMAP before and after batch correction. (e)-(f) Heatmap showing the top 5 discriminative marker genes of each UMAP cluster and cell clusters after annotation. (g) Cell clusters for SA, acne and normal group respectively revealed by UMAP. (h)-(i) The proportion of cell clusters. Fig. S4. Functional enrichment analysis of macrophage-specific DEGs. (a)-(b) GO and KEGG functional enrichment analysis for all macrophage-specific DEGs. (c)-(d) GO and KEGG functional enrichment analysis for up-regulated macrophage -specific DEGs. (e)-(f) GO and KEGG functional enrichment analysis for down-regulated macrophage-specific DEGs. Fig. S5. Expression status of *TIM3*. (a) The symmetrical funnel plot showed the heterogeneity was acceptable in the MR analysis of TIM3. (b) The leave-one-out analysis showed in the MR analysis of TIM3, the estimates were not biased by a single SNP. (c) The expression of *TIM3* as shown through scRNA analysis. (d) The expression of *TIM3* showed through dot plot. Fig. S6. Detailed cell–cell communication inferred from CellChat. (a) The number of interactions and interaction weight/strength between cells inferred from CellChat. (b) Detailed intercellular communications for each cell type. (c) Intercellular communications in galectin signaling network. (d) The heatmap showed the role of each cell type in galectin signaling pathway network. Fig. S7. Detailed GAL9 related pathways. Table S1. The primers for qPCR used in our study.

## Data Availability

The scRNA-seq data for acne and normal were downloaded from the GEO database with an accession number of GSE175817. The scRNA-seq data for SA and normal samples collected in West China Hospital is deposited in China National Center for Bioinformation-National Genomics Data Center (PRJCA051568). Other data that support the findings of this study are available on request from the corresponding author.
