# Supplementary material for: T-cell immunoglobulin and mucin domain-containing protein 3–mediated immunomodulation in myeloid cells and keratinocytes in the development of severe acne
Source: Mol Biomed. 2025 Nov 27;6:125. doi: 10.1186/s43556-025-00367-3 (PMC12660616; doi:10.1186/s43556-025-00367-3)
Supplement: Supplementary file 1 — Supplementary Material 1: Fig. S1. Workflow of the whole study. Fig. S2. Clinical data of SA patients. (a) Clinical data of patients involved in scRNA-seq. (b) Clinical data of patients involved in mIHC. (c)Representative images of SA patients. Fig. S3. scRNA data processing. (a) Violin plot before quality control. (b) Violin plot after filtering cells with the threshold of genes more than 200 and less than 2500, and mitochondrial genes below 5%. (c) Heatmap for the expression profiles of the first 2 principal components. (d) UMAP before and after batch correction. (e)-(f) Heatmap showing the top 5 discriminative marker genes of each UMAP cluster and cell clusters after annotation. (g) Cell clusters for SA, acne and normal group respectively revealed by UMAP. (h)-(i) The proportion of cell clusters. Fig. S4. Functional enrichment analysis of macrophage-specific DEGs. (a)-(b) GO and KEGG functional enrichment analysis for all macrophage-specific DEGs. (c)-(d) GO and KEGG functional enrichment analysis for up-regulated macrophage -specific DEGs. (e)-(f) GO and KEGG functional enrichment analysis for down-regulated macrophage-specific DEGs. Fig. S5. Expression status of TIM3. (a) The symmetrical funnel plot showed the heterogeneity was acceptable in the MR analysis of TIM3. (b) The leave-one-out analysis showed in the MR analysis of TIM3, the estimates were not biased by a single SNP. (c) The expression of TIM3 as shown through scRNA analysis. (d) The expression of TIM3 showed through dot plot. Fig. S6. Detailed cell–cell communication inferred from CellChat. (a) The number of interactions and interaction weight/strength between cells inferred from CellChat. (b) Detailed intercellular communications for each cell type. (c) Intercellular communications in galectin signaling network. (d) The heatmap showed the role of each cell type in galectin signaling pathway network. Fig. S7. Detailed GAL9 related pathways. Table S1. The primers for qPCR used in our study. [file 43556_2025_367_MOESM1_ESM.docx]

**Supporting Information**

1. **cell immunoglobulin and mucin domain-containing protein 3–mediated immunomodulation in myeloid cells and keratinocytes in the development of severe acne**
2. Siliang Chen^1,†^, Xiaoyun Wang^1,†^, Yidan Xu^1^, Wanxin Zeng^1^, Gu He^1,2,*^, Xiang Wen^1,*^

*^1^ Department of Dermatology and Venereology, West China Hospital, Sichuan University, Chengdu 610041, China.*

*^2^ Laboratory of Dermatology, Clinical Institute of Inflammation and Immunology, Frontiers Science Center for Disease-related Molecular Network, State Key Laboratory of Biotherapy, West China Hospital, Sichuan University, Chengdu 610041, China.*

† These authors contributed equally.

* Correspondence:

Gu He

hegu@scu.edu.cn

Xiang Wen

wenxiang@wchscu.cn


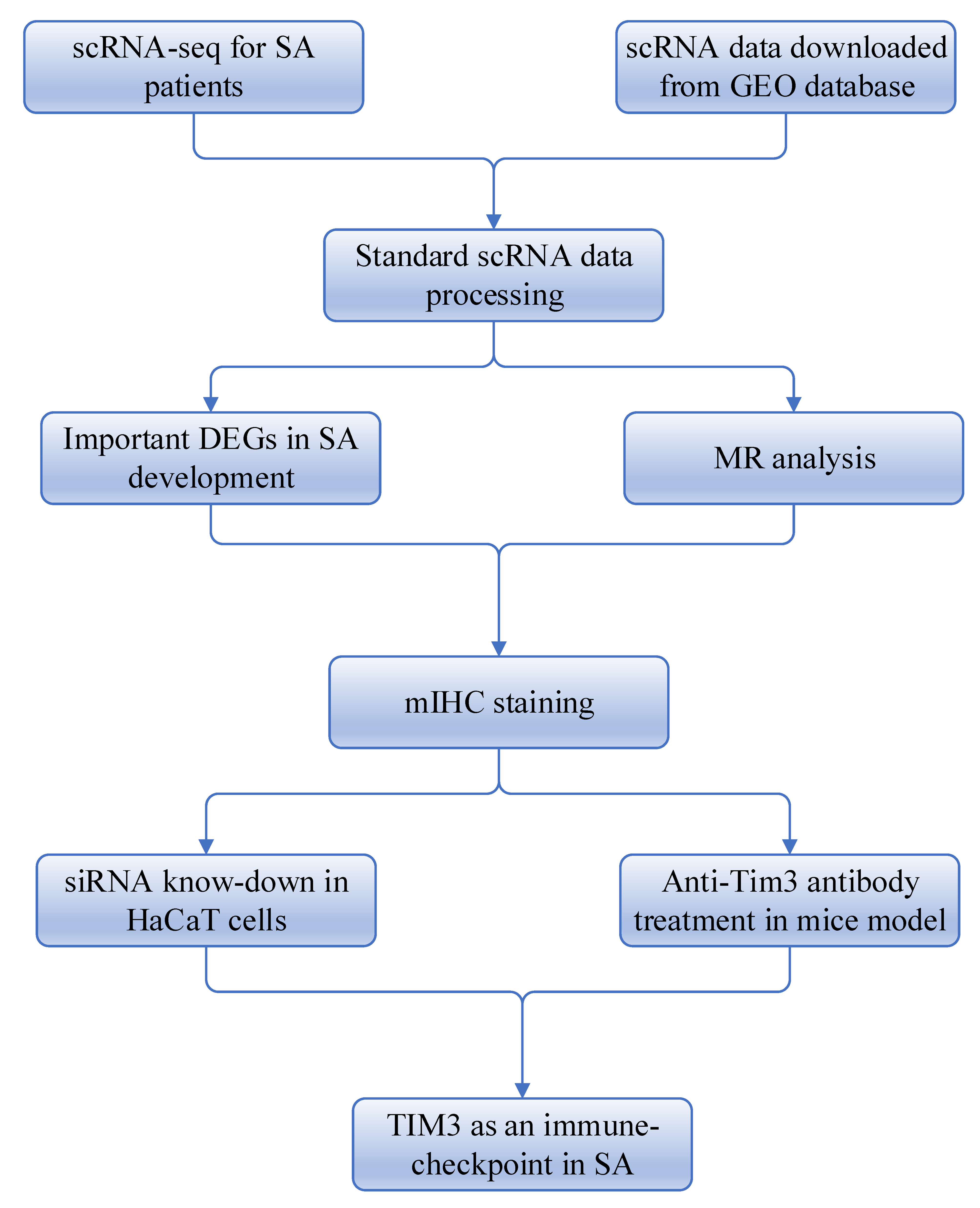


**Figure S1. Workflow of the whole study.**


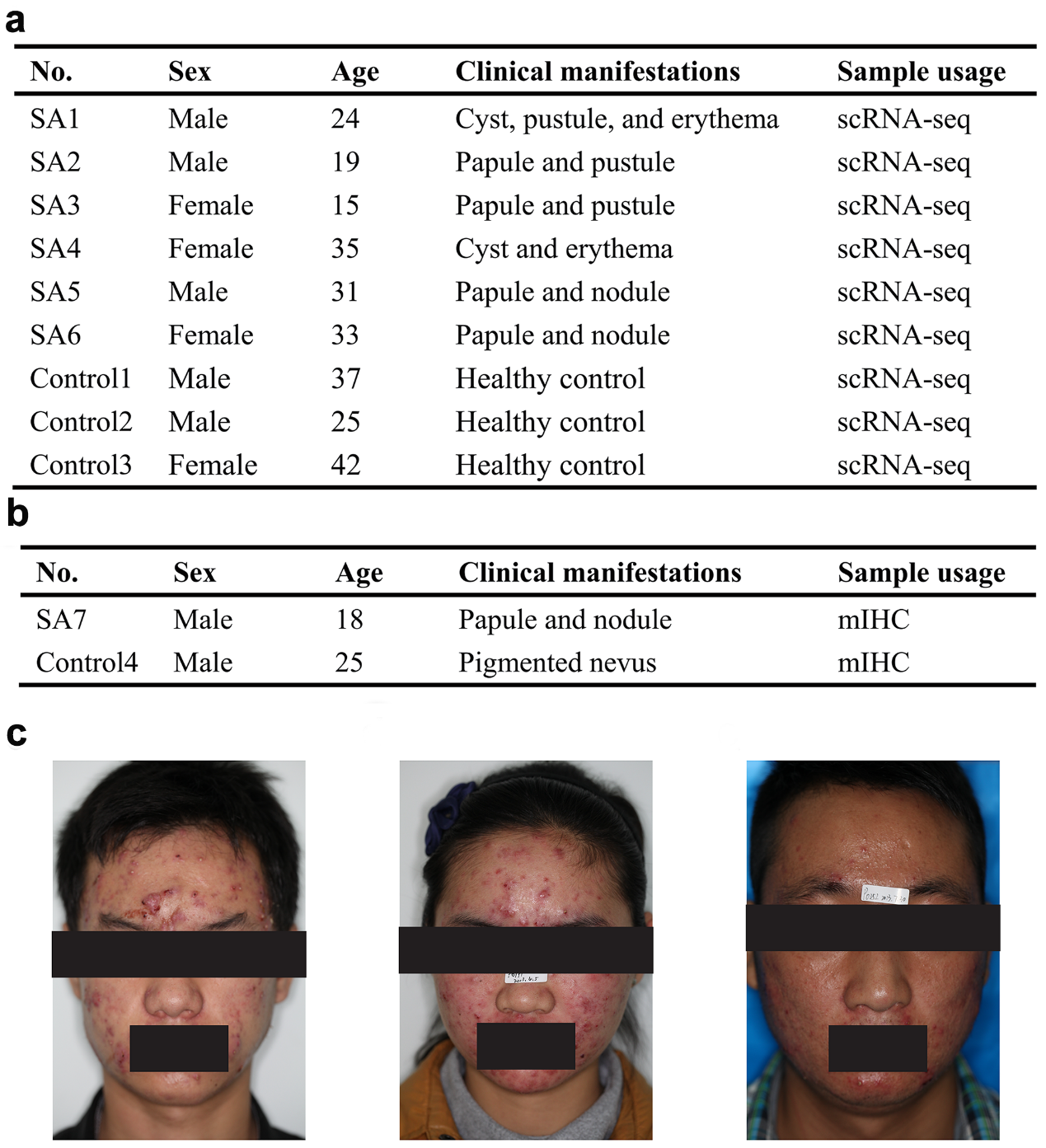


**Figure S2. Clinical data of SA patients.** (a) Clinical data of patients involved in scRNA-seq. (b) Clinical data of patients involved in mIHC. (c) Representative images of SA patients.


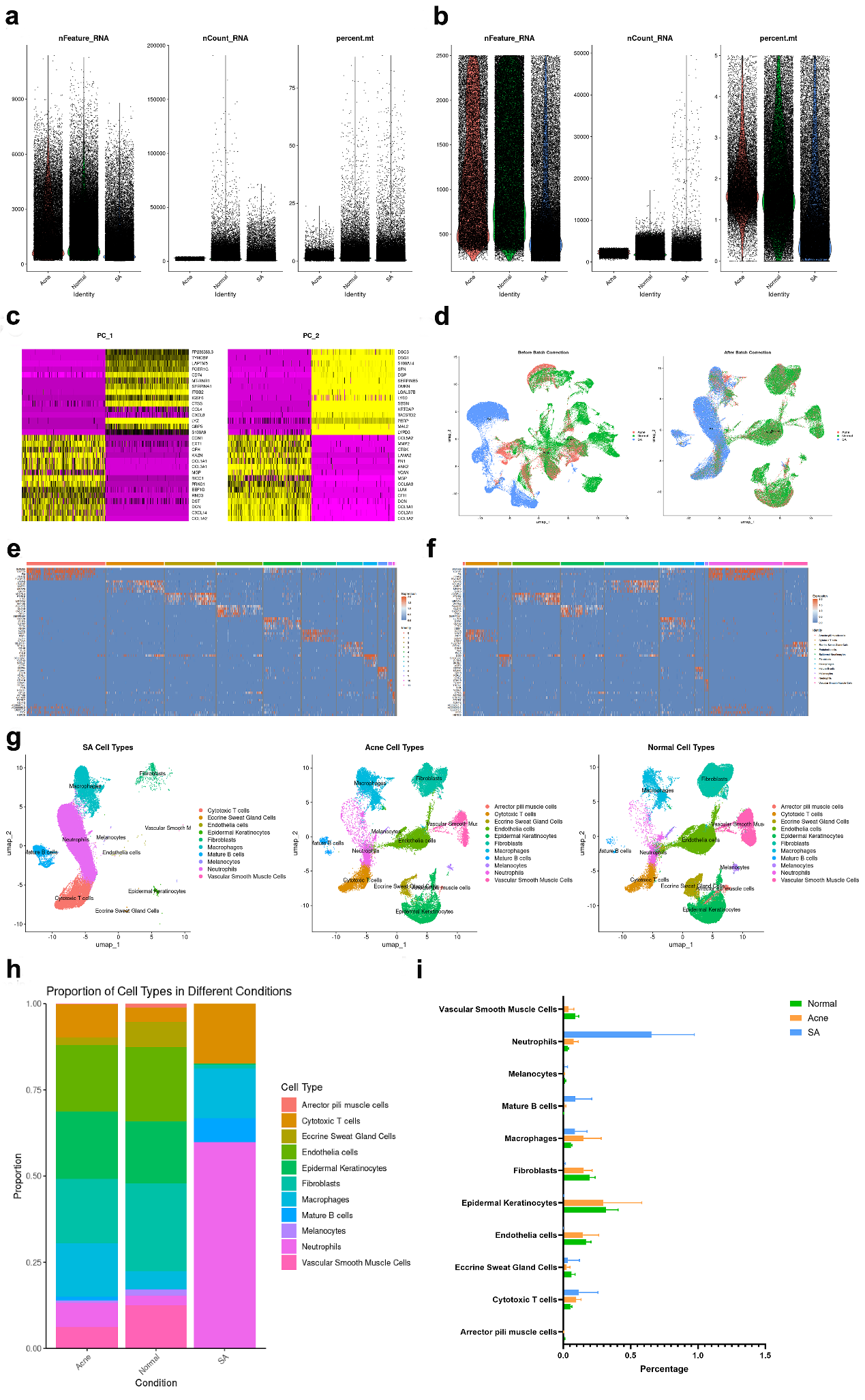


**Figure S3. scRNA data processing.** (a) Violin plot before quality control. (b) Violin plot after filtering cells with the threshold of genes more than 200 and less than 2500, and mitochondrial genes below 5%. (c) Heatmap for the expression profiles of the first 2 principal components. (d) UMAP before and after batch correction. (e)-(f) Heatmap showing the top 5 discriminative marker genes of each UMAP cluster and cell clusters after annotation. (g) Cell clusters for SA, acne and normal group respectively revealed by UMAP. (h)-(i) The proportion of cell clusters.


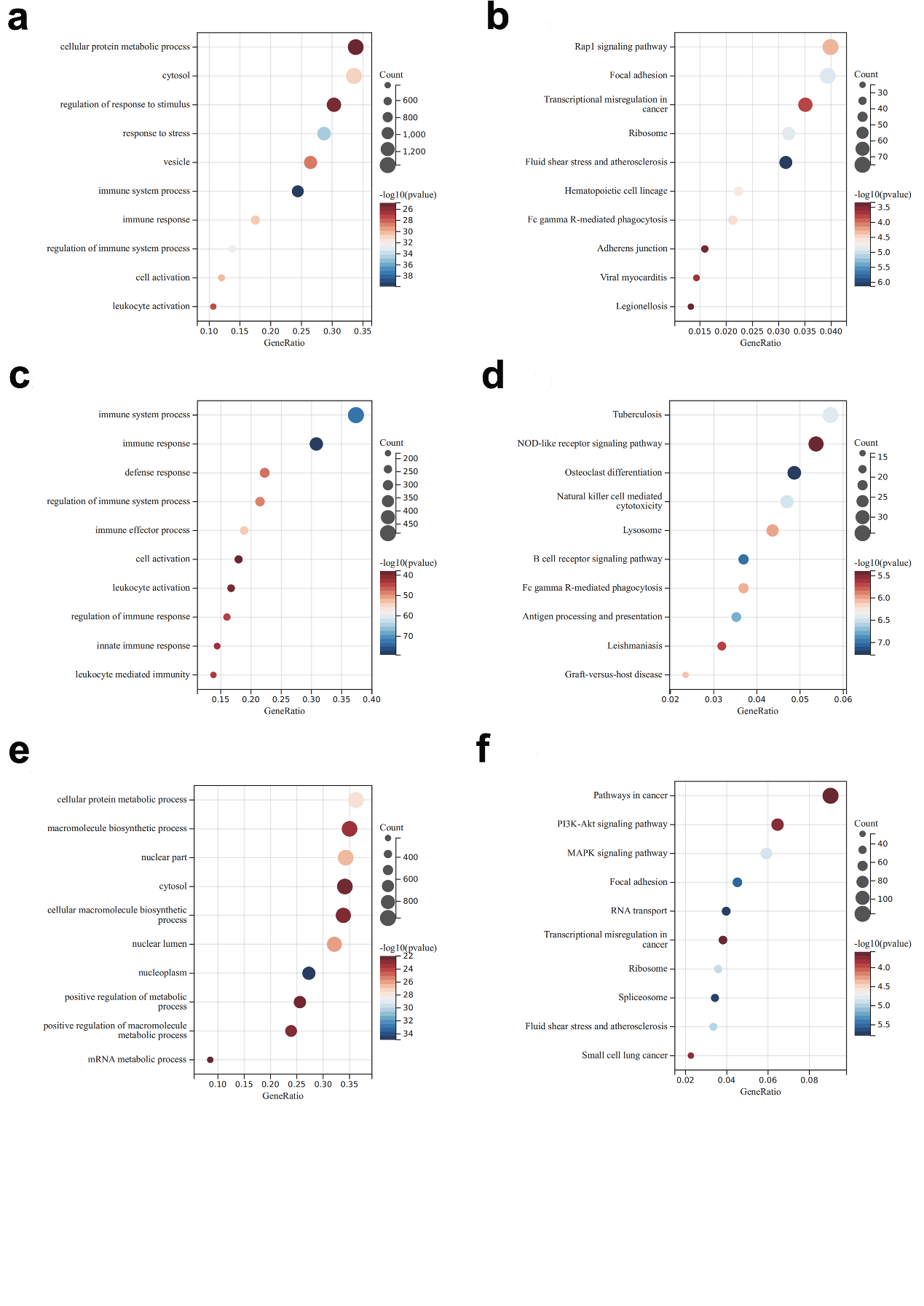


**Figure S4. Functional enrichment analysis of macrophage-specific DEGs.** (a)-(b) GO and KEGG functional enrichment analysis for all macrophage-specific DEGs. (c)-(d) GO and KEGG functional enrichment analysis for up-regulated macrophage-specific DEGs. (e)-(f) GO and KEGG functional enrichment analysis for down-regulated macrophage-specific DEGs.


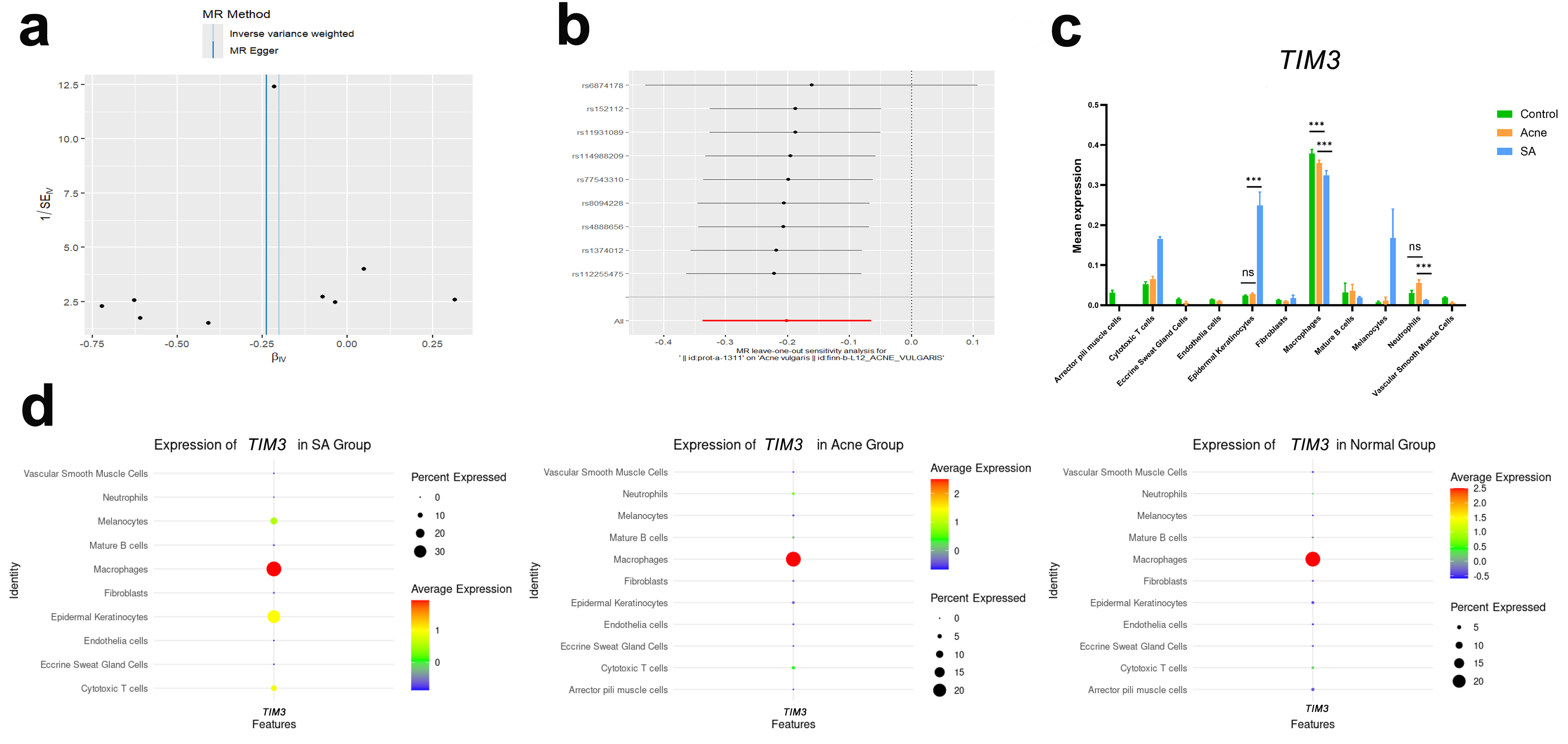


**Figure S5. Expression status of *TIM3*.** (a) The symmetrical funnel plot showed the heterogeneity was acceptable in the MR analysis of TIM3. (b) The leave-one-out analysis showed in the MR analysis of TIM3, the estimates were not biased by a single SNP. (c) The expression of *TIM3* as shown through scRNA analysis. (d) The expression of *TIM3* showed through dot plot.


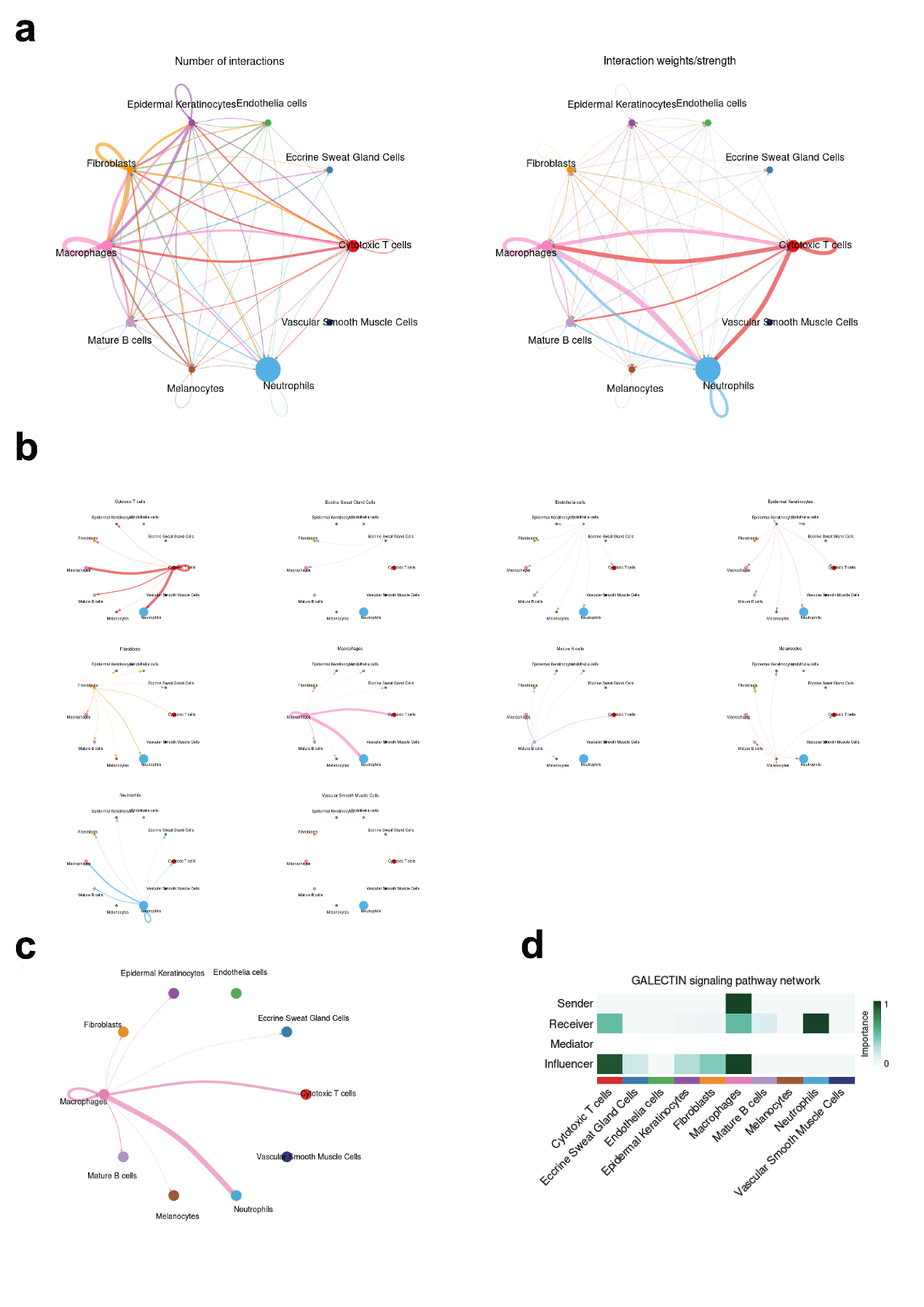


**Figure S6.** **Detailed cell-cell communication inferred from CellChat.** (a) The number of interactions and interaction weight/strength between cells inferred from CellChat. (b) Detailed intercellular communications for each cell type. (c) Intercellular communications in galectin signaling network. (d) The heatmap showed the role of each cell type in galectin signaling pathway network.


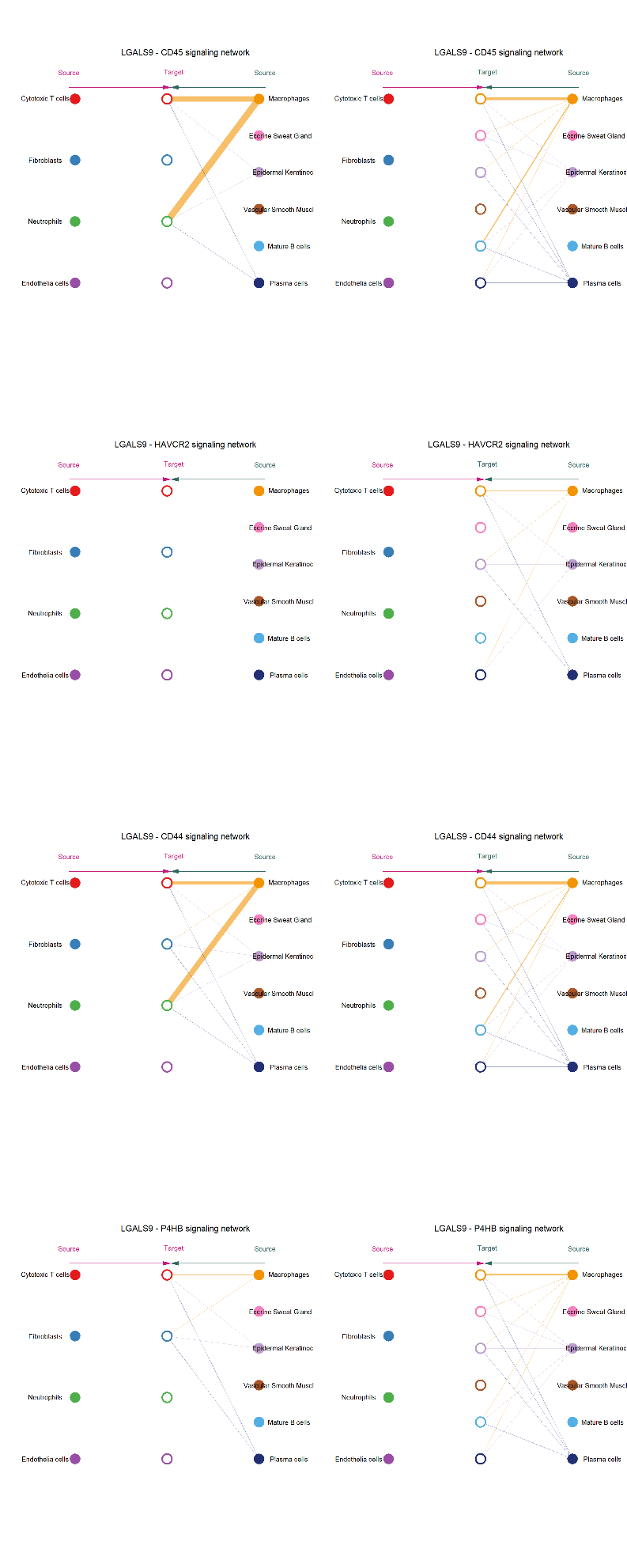


**Figure S7. Detailed GAL9 related pathways.**

**Table S1. The primers for qPCR used in our study.**

| **Genes** |  | **Sequence** |
| --- | --- | --- |
| TIM3 | Forward | 5′-CCTATCTGCCCTGCTTCTAC-3′ |
|  | Reverse | 5′-CTGGTGGTAAGCATCCTTGG-3′ |
| GAL9 | Forward | 5′-GGACGGACTTCAGATCACTGT-3′ |
|  | Reverse | 5′-CCATCTTCAA-ACCGAGGGTTG-3′ |
| IL1β | Forward | 5′-ATGATG-GCTTATTACAGTGGCAA-3′ |
|  | Reverse | 5′-GTCGGAGATTCGTAGCTGGA-3′ |
| IL6 | Forward | 5′-AACCTGAACCTTCCAAAGATG-3′ |
|  | Reverse | 5′-TCTGGCTTGTTCCTCACTACT-3′ |
| IL8 | Forward | 5′-AATCAGTGAAGATGCCAGTG-3′ |
|  | Reverse | 5′-GCACAGTG-GAACAAGGACT-3′ |
| TNF-α | Forward | 5′-CCCCAGGGACCT-CTCTCTAA-3′ |
|  | Reverse | 5′-GCTTGAGG-GTTTGCTACAACA -3′ |
| GAPDH | Forward | 5′-GGTGGTCTCCTCTGACTTCAACAG-3′ |
|  | Reverse | 5′-GTTGTTGTAGCCAAATTCGTTGT-3 ′ |
